# New Adamantane-Containing Edaravone Conjugates as Potential Neuroprotective Agents for ALS Treatments

**DOI:** 10.3390/molecules28227567

**Published:** 2023-11-13

**Authors:** Maria A. Lapshina, Elena F. Shevtsova, Vladimir V. Grigoriev, Aleksey Yu. Aksinenko, Aleksey A. Ustyugov, Daniil A. Steinberg, Grigoriy V. Maleev, Elena S. Dubrovskaya, Tatiana V. Goreva, Tatiana A. Epishina, Vladimir L. Zamoyski, Galina F. Makhaeva, Vladimir P. Fisenko, Ivan M. Veselov, Daria V. Vinogradova, Sergey O. Bachurin

**Affiliations:** 1 Institute of Physiologically Active Compounds at the Federal Research Center of Problems of Chemical Physics and Medicinal Chemistry, Russian Academy of Sciences, 1 Severnij proezd, 142432 Chernogolovka, Russia; lapshina.masha@yandex.ru (M.A.L.); shevtsova@ipac.ac.ru (E.F.S.); grigor@ipac.ac.ru (V.V.G.); alaks@ipac.ac.ru (A.Y.A.); alexey@ipac.ac.ru (A.A.U.); danilashteinberg@yandex.ru (D.A.S.); g.maleev@ipac.ac.ru (G.V.M.); dubrovskaya1250@mail.ru (E.S.D.); tg1960@mail.ru (T.V.G.); tatiana.epishina@mail.ru (T.A.E.); vzam@yandex.ru (V.L.Z.); gmakh@ipac.ac.ru (G.F.M.); jowent@mail.ru (I.M.V.); vindarvik@gmail.com (D.V.V.); 2 Department of Pharmacology, Sechenov I. M. First Moscow State Medical University, 8 Build. 2 Trubetskaya Str., 119991 Moscow, Russia; vpfisenko@mail.ru

**Keywords:** amyotrophic lateral sclerosis (ALS), edaravone, aminoadamantanes, lipid peroxidation, mitochondria, FUS protein, aggregation, fast sodium currents

## Abstract

Currently, there are no effective drugs for the treatment of amyotrophic lateral sclerosis (ALS). Only two drugs—edaravone and riluzole—have been approved, but they have very limited efficacy. The aim of this work was to modify the structural core of the Edaravone—phenylpyrazolone moiety and combine it with aminoadamantane pharmacophore in order to expand the spectrum of its action to a number of processes involved in the pathogenesis of ALS. New conjugates of edaravone derivatives with 1-aminoadamantanes combined with alkylene or hydroxypropylene spacers were synthesized, and their biological activity was investigated. Compounds were found that could inhibit lipid peroxidation and calcium-related mitochondrial permeability, block fast sodium currents of CNS neurons, and reduce aggregation of the mutated form of the FUS-protein typical to ALS. So, the proposed modification of the edaravone molecule has allowed the obtaining of new original structures that combine some prospective therapeutic mechanisms against key chains of the pathogenesis of ALS. The identified lead compounds can be used for further optimization and development of new promising drugs on this basis for the treatment of ALS.

## 1. Introduction

Amyotrophic lateral sclerosis (ALS) is a severe neurodegenerative disease characterized by selective damage to motor neurons, and rapid and steady progression, in most cases leading to death within a few years from the onset [1]. The development of ALS is based on a range of phenomena that lead to the impaired functioning and subsequent complete degeneration of motor neurons. Thus, there is an uncontrolled pathological aggregation of some specific proteins (proteinopathy) [2], an impairment of neurotransmitter processes—in particular, the glutamatergic system [3], lipid peroxidation (LP) membranes of motor neurons and glial cells—as well as a number of other processes [4]. In general, ALS has a multiple nature of pathology development, in which a large number of biological structures are involved, which can be considered as biotargets for potential drugs [5]. Currently, two drugs are used mainly in clinical settings for the treatment of ALS: Riluzole and Edaravone. The former acts on some targets involved in the pathogenesis of ALS, in particular, on fast sodium currents, and a number of others. For the second drug, Edaravone, the detailed mechanism of action has not been determined, although it is assumed that the therapeutic effect of Edaravone may be related to its ability to reduce LP in the membranes of motor neurons and glial cells, observed in the development of ALS [6].

The aim of this work was to modify the structural core of the drug Edaravone in order to introduce new types of activities into the biological properties of the drug that are important in terms of action on key stages of the pathogenesis of ALS. Namely, to block sodium currents, as well as to affect an aggregation of the FUS protein involved in the pathogenesis of hereditary forms of ALS [7]. In this relation, we decided to tailor the phenylpyrazolone moiety of Edaravone with aminoadamantine residues—a structural core of neuroprotective medicine Memantine, which is currently discussed as potential therapeutic agent for ALS treatment [8].

## 2. Results and Discussion

### 2.1. Synthesis and Structure of New Adamantane-Containing Edaravone Conjugates

**Figure 1:**
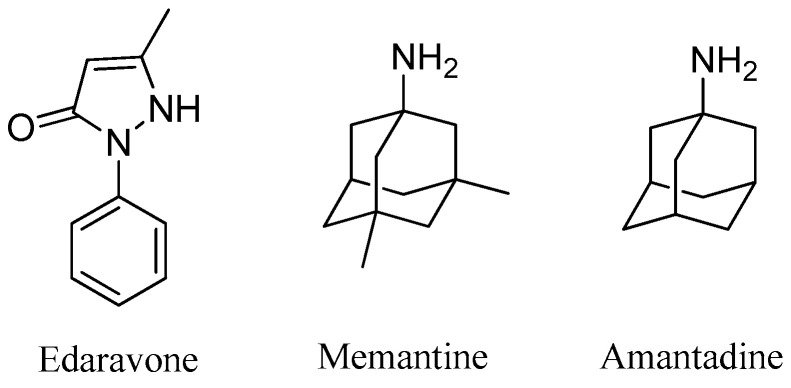
Structures of initial pharmacophores.

A number of original compounds, which are the conjugates of the phenylmethylpyrazolone moiety of Edaravone and the aminoadamantane moieties of Memantine and Amantadine, were synthesized, which effectively block the NMDA subtype of glutamate receptors, as well as its unsubstituted analogue, Amantadine (Figure 1).

**Scheme 1:**
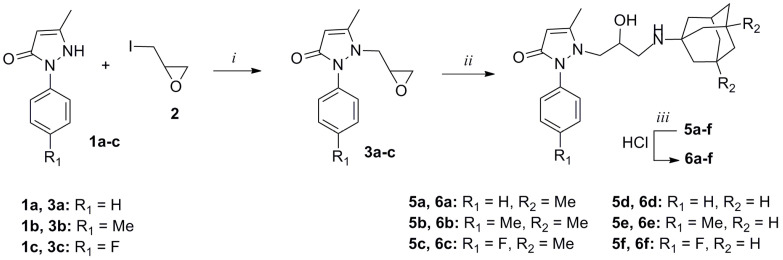
Reagents and conditions: *i*. K_2_CO_3_, DMF, 20 °C, 1 day; *ii*. **4a** or **4b**, BiCl_3_, heptane, 120 °C, 18 h; *iii*. HCl, acetone, 20 °C.

**Scheme 2:**
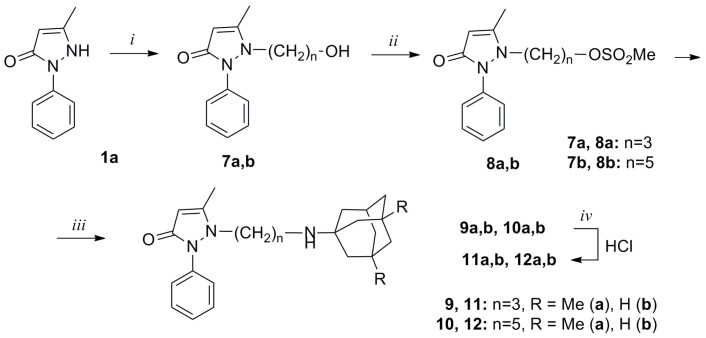
Reagents and conditions: *i*. Br(CH_2_)_n_OH, K_2_CO_3_, DMF, 20 °C, 1 day; *ii*. MeSO_2_Cl, Et_3_N, CHCl_3_, 20 °C, 1 day; *iii*. **4a** or **4b** *i*-PrOH, 120 °C, 15 h; *iv*. HCl, acetone, 20 °C.

In this study, the strategy previously formulated by the authors for the development of multipharmacophore drugs was used [9]. A number of conjugates of phenylmethylpyrazolone derivatives and 1-aminoadamantanes linked via 2-hydroxypropylene or alkylene spacers were synthesized (Scheme 1 and Scheme 2).

The conjugates **6a**–**f** with hydroxypropylene spacer were synthesized by epoxymethylation of pyrazolones **1a**—**c** with epiiodhydrin (**2**) and subsequent opening of the oxirane ring in the resulting pyrazolones **3a**—**c** by the amino group of agents Amantadine (**4a**) and Memantine (**4b**) (Scheme 1).

Compounds **11a**, **11b** and **12a**, **12b** were synthesized by alkylation of pyrazolone **1a** with bromoalcanols in the presence of K_2_CO_3_ and subsequent sulfonylation of the obtained alcohols **7a**, **7b** with methanesulfonyl chloride in the presence of Et_3_N. Further amination of mesylates **7a**, **7b** with an excess of 1-aminoadamantane **4a** and 3,5-dimenthyl-1-adamantane **4b** in *i*-PrOH at 120 °C in a sealed tube led to the target conjugates **9**, **10**, which were converted into the corresponding hydrochlorides **11a**, **11b** and **12a**, **12b** (Scheme 2).

Detailed synthesis and spectral data of compounds **6a**–**f**, **11a**, **11b** and **12a**, **12b** are presented in the article [10].

### 2.2. Investigation of the Effect of Adamantane-Containing Edaravone Conjugates on the LP of Rat-Brain Homogenate

The death of motor neurons in ALS is largely associated with ferroptosis, a programmed cell death characterized by iron- and H_2_O_2_-dependent LP [11]. Therefore, the ability of these compounds to suppress the lipid peroxidation (LP) could be an important feature for neuroprotection of potential drugs with anti-oxidant potential.

**Figure 2:**
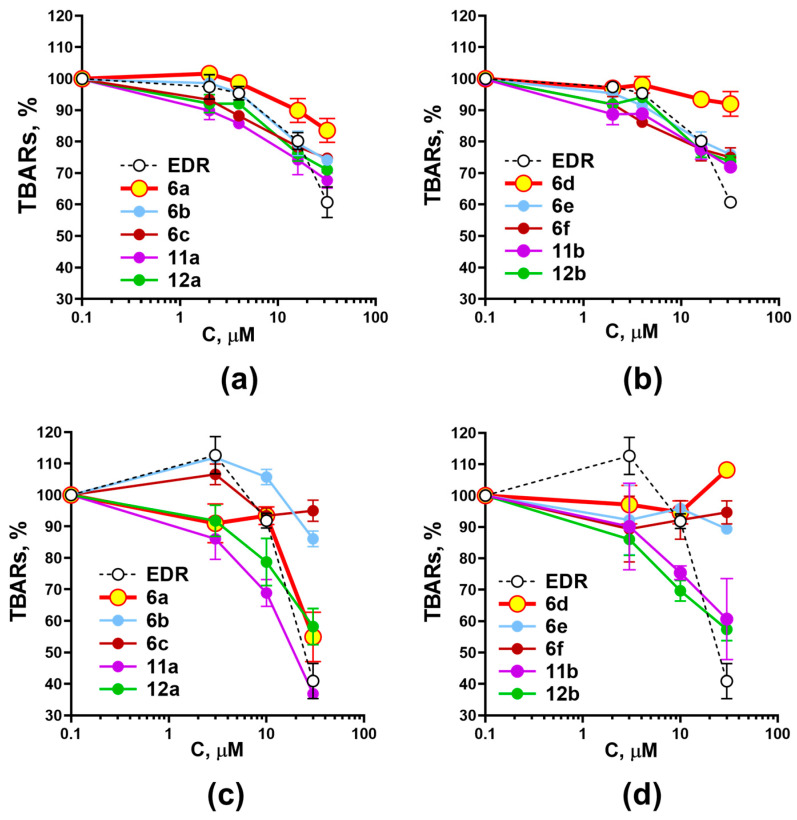
Evaluation of the concentration dependence of the ability of edaravone and its conjugates with 1,3-dimethylaminoadamantane (**a**,**c**) or aminoadamantane (**b**,**d**) to inhibit the development of LP in rat-brain homogenate (2.5 mg of protein/mL) induced by 0.5 mM Fe^2+^ (**a**,**b**) or 10 mM H_2_O_2_ (**c**,**d**). The level of LP was determined by the formation of diene aldehydes, in particular MDA, and their reaction with thiobarbituric acid and they were designated as TBARs (thiobarbituric acid reactive substances). The data were normalized between the probe without any Oxidant (0%) and the probe with oxidant (Fe^2+^ or H_2_O_2_) and buffer + DMSO instead of compound.

The effect of edaravone itself and its new conjugates on the LP of rat-brain homogenate induced by both iron ions and hydrogen peroxide was investigated. The results are presented in Figure 2.

In the case of H_2_O_2_-induced LP (Figure 2c,d), a pronounced dependence of the nature of the inhibitory activity on the structure of the conjugates was established. Thus, for compounds with an alkylene spacer (**11** and **12**), regardless of the substituents in the aminoadamantane moiety, a concentration-dependent inhibition of the LP process is shown, and in the case of the hydroxypropylene spacer (**6**), differences are observed depending on the structure of the aminoadamantane moiety. Memantine conjugates inhibit LP in a concentration-dependent manner (Figure 2c), with the lowest activity for the fluorine-containing conjugate **6c**. At the same time, amantadine conjugates (Figure 2d) do not show the ability to suppress LP up to 30 μM. In the case of Fe^2+^-induced LP (Figure 2a,b), concentration-dependent suppression of LP is also shown, although it is slightly weaker compared to H_2_O_2_-induced LP. However, even in this case, amantadine-containing conjugates exhibit lower activity compared to memantine-containing ones.

For further investigation, conjugates of unsubstituted edaravone with “memantine” 6a and “amantadine” **6d** moieties and a hydroxypropylene spacer were selected, taking into account the ability of **6a** to reduce LP almost equally to edaravone, and the absence of such activity in the case of **6d**.

**Figure 3:**
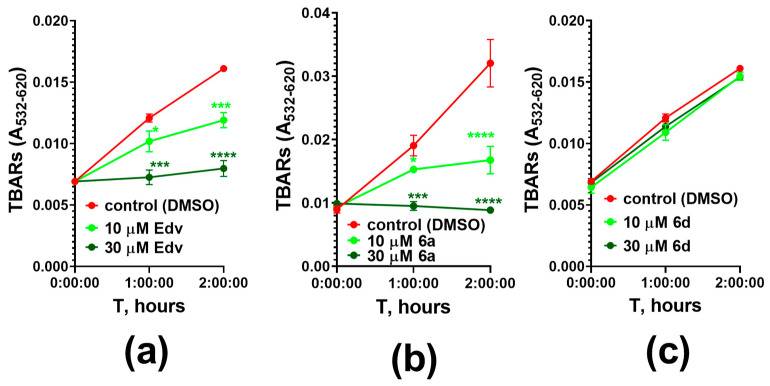
Effect of edaravone (**a**) and conjugates **6a** (**b**) and **6d** (**c**) on LP in rat-brain homogenate, measured by the accumulation of TBARs. The control probe (control (DMSO)) includes the DMSO in the same volume as in the probes with compounds. The significance of differences for each time point was determined by ANOVA analysis with the post-hoc Dunnett’s test for multiple comparisons: *—*p* ≤ 0.05; ***—*p* ≤ 0.001; ****—*p* ≤ 0.0001.

In the study of the effect of the selected compounds on spontaneous LP of the rat-brain homogenate, the anti-oxidant potential of memantine-containing edaravone conjugate 6a, which is not inferior to the activity of edaravone, was confirmed, as shown in Figure 3a and 3b, respectively. At the same time, amantadine-containing analogue **6d**, which does not show inhibitory activity in relation to H_2_O_2_-induced LP is also not able to reduce spontaneous LP in the rat-brain homogenate.

**Figure 4:**
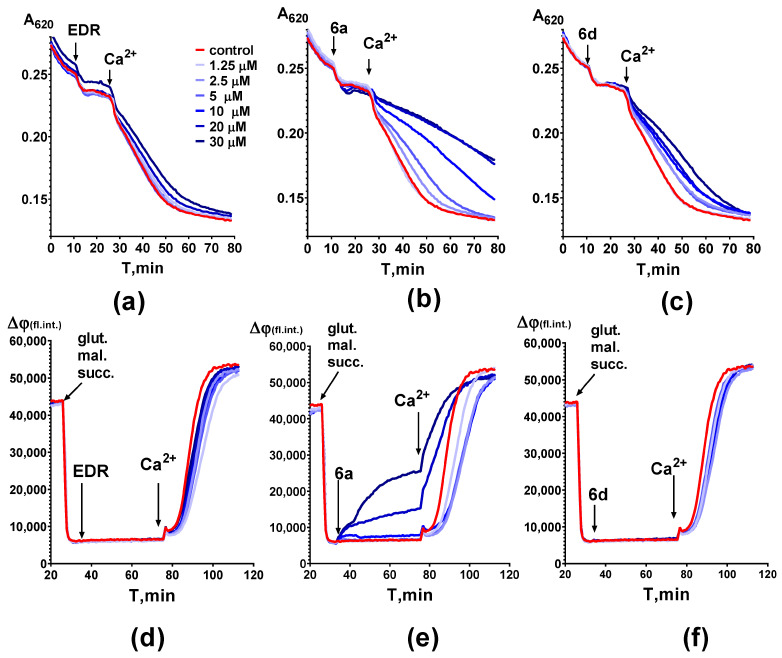
Effect on Ca^2+^-induced mitochondrial swelling (**a**–**c**) and mitochondrial potential (**d**–**f**) of edaravone (**a**,**d**) and conjugates **6a** (**b**,**e**) and **6d** (**c**,**f**). Control probes were injected with the same volume of DMSO instead of compounds. The figure shows the results of one experiment; each curve of the graph represents the average of three individual samples. Three experiments were conducted on different preparations of isolated mitochondria, and no differences were found.

A possible mechanism of LP suppression, including spontaneous LP, may be associated with (due to) the effect of compounds on mitochondrial functions, the decreasing of free radical production, or the preventing of mitochondrial permeability transition (MPT). In this work, we investigated the effect of both edaravone and its conjugates on calcium-induced swelling of rat-liver mitochondria due to MPT, on mitochondrial potential (under conditions of glutamate–malate–succinate-induced energization) and calcium-induced depolarization (Figure 3). For edaravone, we were unable to identify either an effect on the mitochondrial potential or a significant inhibition of the Ca^2+^-induced mitochondrial swelling as marker of MPT (Figure 4a,d), although its ability to reduce the number of swollen mitochondria was previously shown [12]. It should be noted that the effect found by the authors, despite the significance of the differences from the control arising in the presence of edaravone, did not exceed 5% [12].

In our experiments, both conjugates significantly inhibited calcium-induced mitochondrial swelling (Figure 4b,c). For the memantine-containing conjugate **6a**, in contrast to the amantadine-containing **6d**, this effect was accompanied by mitochondrial depolarization (Figure 4e,f). The mechanism of depolarization requires further investigation. However, it is obvious that both compounds may prevent the development of calcium-induced mitochondrial permeability, and it may lead to their neuroprotective effect. Moreover, the memantine-containing conjugate **6a** also has a significant anti-oxidant potential and is able to prevent the development of both spontaneous LP (Figure 3b), and H_2_O_2_-induced LP (Figure 2c).

### 2.3. Investigation of the Effect of Adamantane-Containing Edaravone Conjugates on Fast Sodium Currents

Patients with ALS are characterized by hyperexcitability of neurons in the cerebral cortex and motor neurons. ALS is associated with impaired ion conduction and, in particular, with increased sodium and reduced axonal potassium conductance, which may underlie neurodegeneration and contribute to the development of such symptoms as fasciculations and muscle spasms found in patients with ALS [13,14].

The data obtained with the genetic ALS model also suggest that neuronal hyperexcitability is associated with an increase in sodium currents [15].

It was shown earlier that Edaravone does not manifest this type of activity in contrast to riluzole, the most often used medicine for the treatment of ALS, for which the ability to reduce fast sodium currents is one of the main elements in the mechanism of its therapeutic effect [6,16]. The effect of synthesized conjugates on voltage-gated fast sodium currents in freshly isolated neurons of the mammalian brain was evaluated. It was revealed that the synthesized edaravone conjugates inhibit the fast sodium currents of neurons. The results obtained are presented in Table 1. For comparison, the data obtained for edaravone, riluzole, as well as amantadine and memantine, which are structural components of the corresponding synthesized conjugates, are given.

It can be concluded that most of the studied compounds may suppress fast sodium currents—one of the key processes in the development of ALS—with efficacy equal or even higher than riluzole or memantine itself. The most pronounced effect was revealed for the compounds **6a**, **6b**, **6d**, and **12a**.

### 2.4. Anti-Aggregation Properties of New Conjugates of Edaravone and Aminoadamantane Derivatives

**Figure 5:**
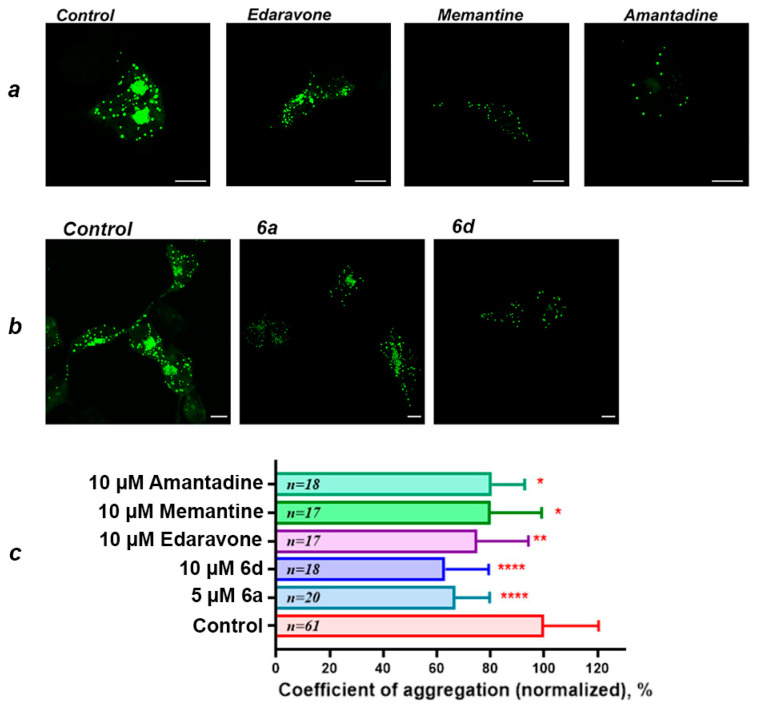
Typical micrographs (scale = 10 μm) of individual SH-SY5Y human neuroblastoma cells transfected by the pEGFP-FUS vector (1-359) after incubation for 24 h (**a**) with model compounds, and (**b**) with conjugates **6a** and **6d**, and (**c**) flow chart with normalized values of FUS protein aggregation coefficients. The data are normalized according to the control sample; the number of cell images analyzed to calculate the aggregation coefficient for each group of compounds is shown in the figure. The significance of the differences was determined by the Kruskal–Wallis test using the post-hoc Dunn’s test for multiple comparisons (****—*p* < 0.0001, **—*p* < 0.01, *—*p* < 0.05).

In this work, we studied the effect of model compounds and new edaravone conjugates **6a** and **6d** on the aggregation of the FUS protein (fused in sarcoma) involved in RNA metabolism, mutations of which were found in patients with ALS. For that purpose, we use a previously developed model system based on SH-SY5Y human neuroblastoma cells, transfected by the pEGFP-FUS vector (1-359) that contain a mutated FUS gene that provides the formation of a protein located exclusively in the cytoplasm [17,18]. An analysis of the dynamics of the formation of cytoplasmic inclusions of the supercoiled protein FUS(1-359) was conducted. The protein FUS(1-359) was selected as the main pathogenic form for modeling “FUSopathy” in SH-SY5Y cell culture since it was the form most susceptible to aggregation and aggression by the attribute [17]. The effect of the tested compounds on the expression and aggregation of the protein FUS(1-359) in human neuroblastoma cells was evaluated using fluorescence confocal microscopy. The results of the action of model compounds and conjugates of edaravone **6d** and **6a** are given in Figure 5. As a control, SH-SY5Y cells transformed by the pEGFP-FUS(1-359) vector not treated with the tested compounds (in the presence of DMSO aliquot) were used. The resulting aggregates in the control culture were differentiated by their large size and great number in comparison with the preparations of cells treated both with model compounds and new conjugates **6a** and **6d**.

Quantitative estimation of compounds’ abilities to reduce the aggregation of the GFP-labeled protein FUS(1-359)—“aggregation coefficients”—were calculated using the method developed earlier to evaluate the anti-aggregation properties of fluorinated gamma-carbolines [18]. The degree of aggregation of the protein FUS(1-359) in the cell cytoplasm and the size of intracellular deposition correlate with the fluorescence intensity: the higher the degree of aggregation of FUS(1-359) protein in the cell cytoplasm, the higher the fluorescence intensity. We carried out a quantitative analysis of the obtained images and calculated the value of the integral intensity of the luminescence of deposits in the cytoplasm of cells treated with the tested compounds, and in control samples not treated with the compounds. The aggregation coefficient was calculated for each compound relative to the untreated control, and reflected the decrease in FUS(1-359) aggregates in the cytoplasm of cells under the action of the tested compounds. The data on the estimated aggregation coefficients for model compounds and compounds **6a** and **6d** are shown in (Figure 5). We have shown that both known model drugs (edaravone, memantine, and amantadine) and new conjugates reduce the aggregation of the protein FUS (1-359), which is a key target for ALS therapy. The model compounds edaravone, memantine, and amantadine possess some anti-aggregant properties (their aggregation coefficients are 75 ± 19%, 80 ± 19%, and 81 ± 12%, respectively). For conjugates **6d** and **6a**, aggregation coefficients are close to each other (63 ± 16% and 67 ± 13%, respectively) and significantly lower than control samples and samples with model compounds. The results obtained permit us to conclude that modification of edaravone with aminoadamantane moieties leads to the manifestation of new anti-aggregation properties.

## 3. Materials and Methods

### 3.1. Investigation of the Effect of Compounds on Lipid Peroxidation in Rat-Brain Homogenate

The anti-oxidant properties of the synthesized conjugates were evaluated by their ability to inhibit lipid peroxidation (LP) in the coarse membrane fraction (1500 g) of the rat-brain homogenate. In the evaluation of spontaneous LP, the brain homogenate in the presence of the studied compound or equal volume of the solvent (DMSO) was incubated for 2 h at 37 °C, taking samples at different time intervals. As for H_2_O_2_ or Fe^2+^ induced LP, the incubation was carried out for 1 h in the presence of 10 mM H_2_O_2_ or 0.5 mM FeSO_4_ × 7H_2_O. The degree of LP was evaluated by the formation of trimethin complexes of secondary products of LP with 2-thiobarbituric acid (TBARs). The experiments were conducted on three different preparations of brain homogenates with three repetitions for each point of individual preparation.

### 3.2. Investigation of the Effect of the Compounds on Mitochondrial Characteristics

Rat-liver mitochondria were isolated by conventional differential centrifugation. Briefly, rats that had been fasted overnight were anesthetized by carbon dioxide and killed by decapitation. The liver was quickly removed and homogenized in an ice-cold isolation buffer (IB)—5 mM HEPES buffer, pH 7.4, containing 210 mM mannitol, 70 mM sucrose, and 1 mM EDTA. The homogenate was centrifuged for 11 min at 1500× *g* (Avanti J-25, Beckmann, San Mateo, CA, USA), and the supernatant was centrifuged at 10,500× *g* for 11 min. A mitochondrial pellet was collected and washed twice using centrifugation. The final pellet was resuspended in IB containing 0.02 mM EGTA instead of EDTA.

The membrane potential of rat-liver mitochondria was measured using the potential-sensitive fluorescent probe Safranine on a EnVision multilabel plate reader (Perkin Elmer, Waltham, MA, USA). The fluorescence was recorded at λex = 485 nm and λem = 590 nm [19]. Mitochondrial swelling was measured by spectrophotometry in 96-well clear bottom plates on an EnVision microplate reader (Perkin Elmer, Waltham, MA, USA) at 620 nm; CaCl_2_ was added to induce mitochondrial swelling. The figure shows the results of one experiment; each curve of the graph represents the average of three individual samples. Three experiments were conducted on different preparations of isolated mitochondria.

Experiments were performed using outbred male rats weighing 200–220 g. The animals were housed under standard vivarium conditions in a normal day/night cycle and had ad libitum access to water and food. All animal manipulations were approved by the local bioethics committee of the Institute of Physiologically Active Compounds of the Russian Academy of Sciences (Protocol No. 41 dated 29 November 2019).

### 3.3. Evaluation of Fast Sodium Currents

The studies were carried out by the electrophysiological method on freshly isolated Purkinje neurons from the cerebellums of Wistar male rats (12–15 days). Single neurons were isolated by the enzyme-mechanical method. Transmembrane currents of individual nerve cells were recorded by the method of local potential fixation (patch-clamp) in the whole-cell configuration using the EPC-9 device (HEKA, Hambrücken, Germany). The data were processed using the Pulsfit program (HEKA, Hambrücken, Germany). To fill the micropipettes, an intrapipette solution was used (120 mM KCl, 2 mM MgSO_4_, 1 mM CaCl_2_, EGTA 11 mM, HEPES 10 mM, K_2_ATP 5 mM, 10 mM glucose, pH = 7.2, osmolarity 295 mOsm). Externally, standard saline solution was used (140 mM NaCl, 5 mM KCl, 2 mM MgSO_4_, 2 mM CaCl_2_, HEPES 10 mM, pH = 7.36, osmolarity 305 mOsm). After the cell membrane breakthrough with a fixation potential of -70 mV on the cell membrane in response to increasing stages of depolarizing pulses of +10 mV, integral ion currents from the whole cell were recorded. The fast input current generated with the MP shift up to +30 mV was a sodium current used to measure and evaluate the efficacy of new compounds.

Compounds were exposed to neurons using the fast perfusion method [20]. Before the first drug application, recordings of fast sodium current were carried out three times and were separated by a minimum of 2 min. The physiological solution in a recording chamber was replaced with the test solutions containing specific concentrations of compounds that were increased throughout the testing period. After each drug application, a 3-min wash-out with physiological solution was carried out and responses to three depolarizing pulses were recorded as a control. The next drug concentration was applied thereafter and was again followed by a wash-out session. The mean of amplitudes of the fast sodium current measured without drugs was taken as a control value, and means of the measurements following the drug applications were normalized to the control values and expressed as a percentage. Normalized currents for control and treatment recordings were obtained for 4–6 cortical neurons, and were analyzed with the specialized software as mentioned above. An IC_50_ for each compound was calculated using the program Statistica, version 13.

### 3.4. Anti-Aggregation Properties of Compounds

The study was carried out on SH-SY5Y human neuroblastoma cells (ATCC, CRL2266, Manassas, VA, USA). The cells were cultured in a modified DMEM/F-12 medium (11320033, Gibco, Grand Island, NY, USA) with the addition of 10% fetal bovine serum, 2 μM glutamine, penicillin (50 U/mL) and streptomycin (50 mg/mL) at 37 °C, atmospheric humidity and 5% CO_2_. The effect of the studied compounds on the aggregation of the FUS protein in SH-SY5Y human neuroblastoma cells was evaluated using fluorescence confocal microscopy, as shown earlier [18]. The cells were seeded into 24-well plates on cover glasses, the density of the cell culture was 6 × 10^4^/mL. After 24 h, temporary transfection of DNA cells with the pEGFPF15 vector was carried out using the TurboFect Transfection Reagent (Thermo Fisher Scientific, R0531, Waltham, MA, USA). Transfection of neuroblastoma cells with a DNA vector was carried out simultaneously with the addition of the tested compounds. After 24 h, the cells were washed from the residues of the medium with 0.01 M phosphate buffer solution PBS (137 mM NaCl, 2.7 mM KCl, pH 7.4) and fixed with a 4% solution of paraformaldehyde for 30 min at room temperature. The aggregation of the aberrant form of the FUS protein conjugated to the GFP (green fluorescence protein) fluorescent label was studied using a laser scanning microscope, Zeiss LSM 880 (Carl Zeiss, Oberkochen, Germany). Images were taken in inverted confocal mode using a C Plan-Apochromat 63×/1.4 Oil DIC M27 lens. A laser with a wavelength of 488 nm was used for excitation, the signal was detected in the wavelength range of 493–600 nm. At least 50 images were analyzed for each studied compound. The degree of aggregation of the FUS protein in the cell cytoplasm and the size of the intracellular deposition correlate with the intensity of fluorescence: the higher the degree of aggregation of the FUS protein in the cell cytoplasm, the higher the intensity of fluorescence. The resulting images were processed using ImageJ image analysis software, version 1.8.0_112, https://imagej.net/ij/ (accessed on 11 September 2023). Quantitative analysis of the obtained images and calculation of aggregation coefficients for control and samples with compounds were conducted according to the formulas, as shown earlier [18], and then normalized, taking the aggregation coefficient in control samples as 100%. Quantitative evaluation and statistical analysis of the degree of FUS protein aggregation in SH-SY5Y cells were carried out using the nonparametric Kruskal–Wallis test with the post-hoc Dunn’s test for multiple comparisons using GraphPad Prism version 7.00 (GraphPad Software, La Jolla, CA, USA).

## 4. Conclusions

In this study it was shown that newly synthesized conjugates of two already known medical agents, edaravone and memantine, exhibit the broad spectrum of properties that could prevent or slow down the development of the neurodegeneration process in ALS. Like edaravone, the conjugates with an alkylene spacer, regardless of the substituents in the aminoadamantane moiety, inhibit Fe^2+^- and H_2_O_2_-induced LP in rat-brain homogenate. In the case of the hydroxypropylene spacer, conjugates of edaravone derivatives and memantine have a greater anti-oxidant activity in relation to H_2_O_2_-induced LP. Edaravone and its memantine-containing conjugate **6a**, in contrast to amantadine conjugate **6d**, also significantly suppress spontaneous LP. Edaravone and its conjugates **6a** and **6d** inhibit calcium-induced “swelling”, which may indicate suppression of the MPT—the key stage in cell death cascade reactions. For memantine-containing conjugate **6a**, in contrast to amantadine-containing **6d**, this effect is accompanied by mitochondrial depolarization and suppression of calcium-induced mitochondrial permeability that may reflect the lack of potential-dependent calcium uptake by mitochondria. Unlike edaravone and amantadine itself, which do not block notably fast sodium currents in the neuronal cell membrane, edaravone conjugates with aminoadamantane derivatives act as effective blockers of these currents, with IC_50_ values close or lower than those of memantine and even riluzole. Of special interest are the results of anti-aggregation properties of novel conjugates. Earlier, it was shown that mutations in genes such as TARDBP, FUS, and HNRNPA1 play important role in the pathogenesis of ALS [21,22]. In particular, the mutant FUS protein can disrupt RNA function and metabolism, proteostasis, or axonal dynamics of the cytoskeleton due to the formation of toxic protein aggregates [23]. Immunoreactivity to the FUS protein is found in cellular inclusions of patients with both sporadic and hereditary ALS, but not associated with SOD1 mutations [24]. Sporadic ALS occurs in the vast majority of patients, but its pathogenetic mechanisms are not known. Therefore, the evaluation of the effects of new compounds on FUS-related pathology is of great interest. The accumulation of pathogenic protein deposits in the cells of the brain and spinal cord in neurodegenerative diseases, including ALS, is used as a marker value to characterize the stages of proteinopathy and directly correlates with the severity of the pathological manifestations of the disease [25]. In this work, for the first time, it was shown that new conjugates of edaravone and aminoadamantane reduce the pathological aggregation of the FUS protein in nerve cells. Thus, the proposed modification of the edaravone molecule has allowed us to obtain new promising compounds that combine the therapeutic potential of edaravone with additional action on some other key chains in ALS pathogenesis. Further optimization of hit-structures can be conducted for the development of a new generation of prospective multi-target drugs for the ALS treatment.

## Figures and Tables

**Table 1:** Effect of novel conjugates and reference agents on fast sodium currents in rat-brain neurons.

Substances	Effect on Fast Na-Currents IC_50_, μM	Substances	Effect on Fast Na-Currents IC_50_, μM
**6a** 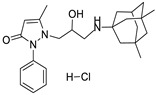	7.8 ± 1.1	**6d** 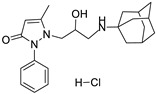	6.9 ± 1.5
**6b** 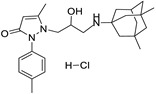	7.1 ± 1.1	**6e** 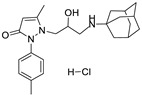	10.9 ± 1.3
**6c** 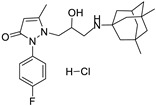	17.2 ± 1.6	**6f** 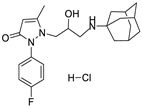	9.5 ± 1.0
**11a** 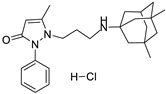	11.8 ± 1.2	**11b** 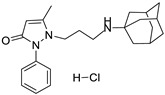	16.2 ± 1.3
**12a** 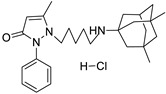	5.9 ± 0.8	**12b** 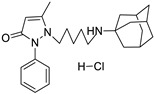	9.9 ± 1.1
	Reference agents		
**Memantine** 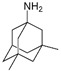	12.8 ± 1.7	**Amantadine** 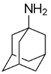	>100
**Edaravone** 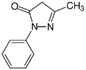	>100	**Riluzole** 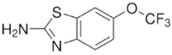	15.3 ± 1.3

## Data Availability

Data are contained within the article.
